# Supplementary material for: HiMoRNA: A Comprehensive Database of Human lncRNAs Involved in Genome-Wide Epigenetic Regulation
Source: Noncoding RNA. 2022 Feb 8;8(1):18. doi: 10.3390/ncrna8010018 (PMC8876941; doi:10.3390/ncrna8010018)
Supplement: Supplementary file 1 [file ncrna-08-00018-s001.zip › ncrna-1473226-supplementary.pdf]

Supplementary Materials

Table S1. An overview of the histone modification and expression data used in HiMoRNA.

Histone Modification	Samples	Expressed lncRNAs
H3K27ac	49	5974
H3K27me3	50	6110
H3K36me3	52	6071
H3K4me1	51	6156
H3K4me2	19	3027
H3K4me3	59	6434
H3K9ac	19	3268
H3K9me3	50	3200
H3K79me2	20	5983
H4K20me1	19	3027

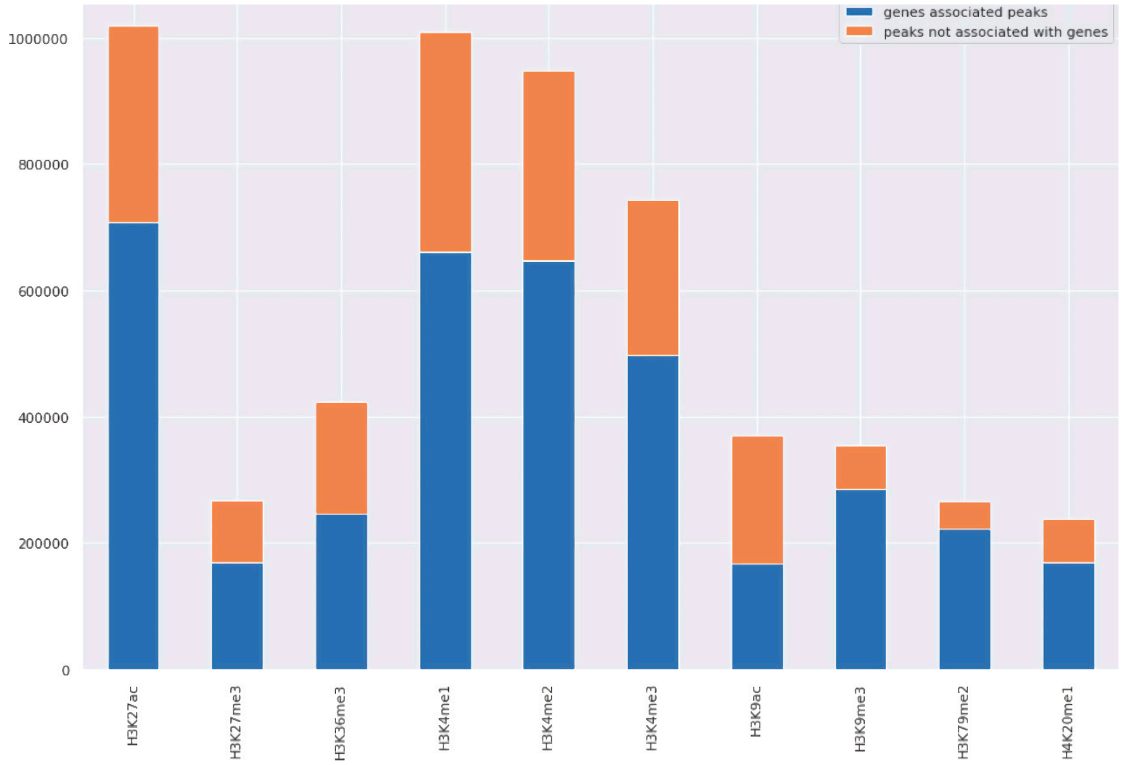

Supplementary Figure S1. Genes to histone modification peaks association.
